# Mental Health and Community Resilience among Vulnerable Populations Affected by Natural Hazards: Protocol for Scoping Reviews

**DOI:** 10.3390/mps5060088

**Published:** 2022-10-28

**Authors:** Shelly Makleff, Karan Varshney, Revathi N. Krishna, Lorena Romero, Jane Fisher

**Affiliations:** 1 Global and Women’s Health Unit, Public Health and Preventive Medicine, Monash University, Level 4, 553 St Kilda Rd, Melbourne, VIC 3004, Australia; 2 Alfred Health, 55 Commercial Road, Melbourne, VIC 3004, Australia

**Keywords:** natural hazard, disaster, vulnerability, mental health, resilience, bushfires, wildfires, drought

## Abstract

Introduction: Exposure to natural hazards such as fire, drought, floods, and earthquakes can have negative impacts on physical and mental health and wellbeing. The social and structural factors contributing to individual and community vulnerability also influence responses to disaster and the resulting consequences on health and wellbeing. Experiencing disasters like bushfires amplifies the impacts of inequality, magnifying existing disparities and contributing to additional psychological burdens of grief, trauma and adaptive challenge. There is a need to understand how vulnerability can influence responses to disaster, and to identify factors that develop and foster resilience in the context of increasing disasters and vulnerability. Materials and Methods: This protocol will describe the methodology of two scoping reviews: the first will describe the mental health outcomes of vulnerable populations after droughts and bushfires; the second will identify and describe strategies that promote community resilience in vulnerable populations in the context of a disaster. A thorough search will be conducted in relevant databases. Studies will be limited to English language. The reviews will be reported using the 22-item checklist for the Preferred Reporting Items for Systematic Reviews and Meta-Analyses. Methodological quality of the included papers will be assessed using the Joanna Briggs Institute’s critical appraisal tools. Results & Conclusions: The two scoping reviews described in this protocol will have broad relevance in the context of increasing and intensifying disasters, and will especially consider the compounded impact of disaster on vulnerable communities. Findings will contribute directly to the design and implementation of solutions to improve post-disaster health and wellbeing and community resilience.

## 1. Introduction

Fires, drought, floods, and earthquakes are among the many types of natural hazards with implications for human health and wellbeing globally. Frequency and intensity of these hazards are increasing around the world, causing increased injury, food shortages, population movements, and elevated risk of contracting communicable diseases [1,2,3]. Exposure to natural hazards can be stressful and potentially traumatising, with impacts on not only physical but also mental health and wellbeing, with outcomes such as post-traumatic stress disorder, distress, anxiety, maladjustment, grief, despair, and hopelessness [4].

Whether a natural hazard event results in a disaster situation for individuals and communities depends on the social and structural factors contributing to that vulnerability. Blaikie and colleagues [5] state, “to understand disasters we must not only know about the types of hazards that might affect people, but also the different levels of vulnerability of different groups of people”. The World Health Organization Commission on the Social Determinants of Health (CSDH) emphasizes that inequalities in health within and among countries are attributable predominantly to differences in the circumstances in which people live [6]. Structural factors including socio-economic policies and distribution of services, and social factors such as access to income-generating work, adequate housing, personal safety, education and transportation, influence inequality and play a role in determining how individuals and communities respond to disaster and the resulting consequences on health and wellbeing. Experiencing disasters like bushfires amplifies the impacts of inequality, further magnifying existing disparities and contributing to additional psychological burdens of grief, trauma and adaptive challenge [4,7]. Some groups are “differentially vulnerable and also differentially resilient in the face of disasters, depending upon their position in the stratification system”, as illustrated in examples around the world ranging from the racial and socio-economic predictors of the negative impacts of Hurricane Katrina in the United States [8] to differential rates of drought mortality among vulnerable groups in Brazil [9]. There remains much to be learned about the influence of individual and community vulnerability on not only the effects of hazards, but also recovery processes.

When confronted with the psychological effects of natural hazards, resilience is a vital protective factor that can improve one’s capability to face such difficulties [4]. There is a growing awareness that successful post-hazard recovery requires resilience at both community and individual levels [10,11]. Consequently, there is a clear need to identify factors that develop and foster resilience in disaster recovery and to understand how resilience to natural hazards relates to mental health and wellbeing, especially in the context of vulnerability [12].

The Fire to Flourish program (2021–2026) in Australia is a longitudinal multi-community program that develops and trials new methods and models of community-led disaster recovery and resilience building in bushfire-affected communities experiencing structural disadvantage [13]. The program was designed in the wake of the Black Summer bushfires in 2019–2020, in which wildfire hazard exposure was associated with socio-economic disadvantage [14]. As an action-based program, Fire to Flourish aims to reduce disadvantage and improve individual and community capabilities, resilience, health and wellbeing. The 5-year program will evolve in response to community needs and priorities and as lessons from early discovery and testing inform refined approaches and provide a foundation for scaling impacts. The program has a significant focus on supporting communities to lead their own local initiatives and connect with each other to create the capacity, conditions and solutions for their long-term resilience. The underlying principles for the program are: be community-led; foreground Aboriginal wisdom; address inequities, enhance inclusion and self-determination; be strengths-based and trauma-informed; be holistic and impactful; learn, adapt and evolve.

The Fire to Flourish program aims to be grounded in evidence, and as part of this focus, aims to identify and build on the existing evidence at the intersection of natural hazards, health and wellbeing, and vulnerability. Scoping reviews are suited to identify evidence and analyze knowledge gaps, with potential to inform program and research development [15]. This protocol details the scoping reviews of the empirical literature that will be carried out as part of the Fire to Flourish program research activities. The objectives of these scoping reviews are to describe the mental health outcomes of vulnerable populations after droughts and bushfires (scoping review 1); and identify and describe strategies that promote community resilience in vulnerable populations in the context of a disaster (scoping review 2).

## 2. Materials and Methods

The design of this protocol has been guided by the Preferred Reported Items for Systematic Review and Meta-Analysis for Protocol (PRISMA-P) [16]. The PRISMA-P checklist is included in Table S1.

A scoping review approach will examine available evidence at the intersection of three bodies of literature: natural hazards, mental health, and vulnerability. Drawing on existing classifications of vulnerability factors relevant to health [17], for the purposes of these scoping reviews we define vulnerable populations as racial/ethnic minorities, elderly individuals, the socioeconomically disadvantaged, those with medical conditions, widows, gender/sexual minorities, and those residing in rural/remote settings.

### 2.1. Search Strategy

Following the development and piloting of the search strategy, the final searches will be conducted by an experienced research librarian on four databases: Ovid Medline (Medical Literature Analysis and Retrieval System Online), Ovid EMBASE (Excerpta Medica Database) CINAHL (Cumulative Index to Nursing and Allied Health Literature), and Ovid PsycInfo (APA PsycINFO). The search strategy will use a combination of database specific subject headings and free text terms that will cover three concept areas: Concept A Bushfires, Wildfires & Natural Disasters; Concept B Mental Health and Wellbeing; and Concept C Disadvantaged and Vulnerable Populations. Furthermore, we will include search terms that cover qualitative methods such as grounded theory, focus groups, phenomenology, and surveys & interviews; and quantitative methods such as cohort and cross-sectional studies. In addition, we will undertake forwards and backwards citation tracking. There are no restrictions on date of publication, however, studies will be limited to English language. The MEDLINE (Ovid) search strategy is provided in Table S2.

### 2.2. Selection of Articles

The search strategy defined above will be the basis for selecting studies for two scoping reviews that support the research aims of the Fire to Flourish program. These reviews will address two interrelated research questions:

Scoping review 1: What are the mental health outcomes for vulnerable adults after a bushfire or drought?
Scoping review 2: What strategies promote community resilience in vulnerable populations in the context of a disaster?

The common selection criteria to identify relevant studies for both scoping reviews are as follows:

- Study type: Peer-reviewed primary research studies (quantitative, qualitative, and mixed methods). Review articles, commentaries, reports, dissertations, conference proceedings, and policy briefs are excluded.
- Population: Studies examining one or more of the following vulnerable groups: racial/ethnic minorities, elderly individuals, the socioeconomically disadvantaged, those with medical conditions, widows, gender/sexual minorities, those residing in rural/remote settings. This excludes studies about well-resourced communities without any reference to these forms of vulnerability.
- Setting: No restrictions on settings (high, low- or middle-income countries).
- Publication timing: No restrictions related to time of publication.
- Language: Only English language articles.

In addition, each scoping review has additional selection criteria.

Scoping review 1:

- Must describe mental health outcome(s) explicitly
- Must be related to experiences of drought or bushfire/wildfire
- Must have adult participants. Excludes studies that focus only on children (under 18 years of age).

Scoping review 2:

- Must mention resilience or one if its dimensions (preparedness, references to coping, adapting, transforming).
- Discusses any type of natural hazard. Other kinds of disaster (technological, human-made, terrorist attacks, gas explosion, pandemics) are excluded.

For each review, we will follow the 22-item checklist for the Preferred Reporting Items for Systematic Reviews and Meta-Analyses (PRISMA-ScR) [18]. Duplicate references will be removed prior to screening. Thereafter, articles will be screened by title/abstract, and full text will then be screening based on the inclusion and exclusion criteria. Full text review will be conducted by two independent reviewers and any conflicts will be resolved via discussion. Covidence will be used for the purpose of screening [19]. Methodological quality of the included papers will be assessed using the Joanna Briggs Institute’s critical appraisal tools [20].

### 2.3. Data Extraction

For each scoping review, two reviewers will independently extract data from the studies that are deemed to be eligible for inclusion after full-text screening. A third reviewer will compare the two sets of extracted data and follow up on any that do not correspond, to reach consensus. A data extraction form will be developed in Covidence. The form will be piloted by at least two independent reviewers for approximately five articles and modified as needed based on team discussion.

The details to be extracted for both scoping reviews include:

- Bibliometric information: title, year of publication, journal, etc.
- Study information: year of data collection, objective, study design, study setting, study limitations
- Study population: participant demographics, vulnerabilities reported, sample size, proportion of the population that is vulnerable
- Natural hazard: type of hazard, year of hazard event

For scoping review 1, additional details to be extracted include focus on individual-level mental health outcomes:

- Mental health outcome(s) assessed
- Measures used to assess mental health outcomes
- Mental health outcomes reported amongst the study population overall, and for vulnerable participants
- Any link (implicit or explicit) between mental health and vulnerability stated by the authors

For scoping review 2, additional details to be extracted focus on community-level resilience:

- Definition of community resilience
- Facilitators and barriers related to disaster recovery
- Strategies promoting community resilience
- Facilitators of community resilience
- Barriers to community resilience
- Measures of community resilience (if used)

### 2.4. Collating, Summarizing, and Reporting

For each review, characteristics related to bibliometric information, study information, study population, and natural hazard will be summarized in a frequency table. Extracted information related to mental health outcomes (scoping review 1) and community resilience (scoping review 2) will be collated, summarized, and reported in tables. Consistent findings across studies, as well as infrequent findings, will be analyzed and discussed in detail. Qualitative analysis will also be conducted when necessary to report pertinent findings relating to study and participant characteristics.

### 2.5. Quality Assessment

All included studies will be assessed for methodological quality using the Joanna Briggs Institute (JBI) critical appraisal tools [20]. As has been conducted in other reviews [21,22], the tools will be altered to provide a numeric score based on the total number of yes/no responses for the metrics of the checklist. Based on the specific JBI critical appraisal tools for each study design, qualitative studies will be assessed on a ten-item scale, cohort studies on an eleven-item scale, and cross-sectional studies will be assessed on an eight-item scale. Quality assessment scores will be compared across studies, and mean assessment scores by study design will be analyzed. Common limitations of included studies will be discussed. These analyses will provide information on the general quality of evidence of the existing literature and highlight areas of weakness that could be a focus in future research.

### 2.6. Ethics and Dissemination

Ethics approval for the secondary data analysis conducted for these scoping reviews is not required. The scoping review results will be made available in peer-reviewed publications, policy briefs and lay-language summaries and presented in conferences and through relevant media outlets. Additionally, the scoping review findings will be presented to communities participating in the Fire to Flourish program and will guide the development of further research and program activities.

## 3. Expected Results and Implications

The two scoping reviews described in this protocol will offer important insights about the literature at the intersection of disaster recovery, vulnerability, and mental health and wellbeing. The reviews will summarize the available evidence regarding the mental health outcomes of fire and drought among vulnerable communities (scoping review 1) and strategies to promote community resilience in vulnerable populations with lived experience of disaster and disadvantage (scoping review 2). Findings from both reviews will have broad relevance, especially in the context of predictions that disasters will become increasingly common and more intense [23,24] and a further understanding that disaster has a compounded impact on vulnerable communities [14]. 

## 4. Conclusions

Findings from these scoping reviews will contribute directly to the design and implementation of solutions to improve post-disaster health and wellbeing and community resilience, particularly in the rural Australian communities engaged in the Fire to Flourish program [13]. Results relating to mental health outcomes will be interpreted in light of existing structural barriers to accessing mental health services, such as stigma and limited availability of service providers, particularly in rural areas [25,26]. Results from the review focusing on strategies promoting community resilience pertaining to all hazards will facilitate response to the increased risk of exposure to multiple disasters among communities facing hardship in Australia and beyond [27,28,29]. The findings of both these scoping reviews will provide context to help understand the mental health impacts and factors that promote community resilience in some of Australia’s most vulnerable communities that have been impacted by the 2019/20 bushfires. This body of knowledge will help programs such as Fire to Flourish respond to the urgent need to build communities’ resilience to disasters and help disrupt the structural disadvantage cycle.

## Data Availability

Not applicable.

## Supplementary Materials

The following supporting information can be downloaded at: https://www.mdpi.com/article/10.3390/mps5060088/s1, Table S1: PRISMA-P (Preferred Reporting Items for Systematic review and Meta-Analysis Protocols) 2015 checklist: recommended items to address in a systematic review protocol* title; Table S2: Ovid MEDLINE(R) and Epub Ahead of Print, In-Process, In-Data-Review & Other Non-Indexed Citations, Daily and Versions(R) Search Strategy.
