# Supplementary material for: Mental Health and Community Resilience among Vulnerable Populations Affected by Natural Hazards: Protocol for Scoping Reviews
Source: Methods Protoc. 2022 Oct 28;5(6):88. doi: 10.3390/mps5060088 (PMC9680364; doi:10.3390/mps5060088)
Supplement: Supplementary file 1 [file mps-05-00088-s001.zip › Table S2.pdf]

Table S2. Ovid MEDLINE(R) and Epub Ahead of Print, In-Process, In-Data-Review & Other Non-Indexed Citations, Daily and Versions(R) Search Strategy

#	Searches
1	disasters/ or natural disasters/ or avalanches/ or cyclonic storms/ or droughts/ or earthquakes/ or floods/ or landslides/ or tidal waves/ or tornadoes/ or wildfires/ or fires/ or Tsunamis/ or Disaster Victims/
2	(Bushfire* or bush fire* or wildfire* or wild fire* or Forest fire* or wildland fire* or wild land fire* or woodland fire* or wood land fire* or brushfire* or brush fire? or rural fire? or grassfire* or grass fire or vegetation fire? or disaster* or flooding or floods or earthquake* or earth quake* or hurricane* or tsunami* or cyclone* or typhoon* or drought? or Tornado* or avalanche* or landslides or volcanic eruption* or natural hazards or natural catastrophe* or ((disast* or catastroph*) adj (fire? or flood or tidal wave? or mudslide* or rockslide* or peatland fire* or peat fire* or storm* or blizzard*))).mp.
3	(postearthquake or postdisaster* or postflood* or post-flood or (Flood adj (related or experience* or exposure or trauma* or impact* or survivor*))).mp.
4	1 or 2 or 3
5	exp mental disorders/ or exp anxiety disorders/ or exp mood disorders/ or neurotic disorders/ or exp "trauma and stressor related disorders"/ or exp mental health services/ or exp counseling/ or Mental Health/ or Anxiety/ or Depression/ or Depressive Disorder/ or Adaptation, Psychological/ or Resilience, Psychological/ or Stress, Psychological/ or Stress Disorders, Post-traumatic/ or Suicide/ or Psychiatric Status Rating Scales/ or Psychological Trauma/ or psychological phenomena/ or posttraumatic growth, psychological/ or emotional adjustment/ or survivorship/ or Survivors/px
6	(depressive* or mental* or post-traumatic stress* or Posttraumatic stress* or PTSD or PTSS or depression or anxiety or resilien* or coping or psych* or wellbeing or well-being or distress* or suicid* or self-harm or schizophreni* or mood disorder* or despair* or counsel?ing or (Posttraumatic growth or Post-traumatic growth)).mp.
7	(((emotional or behavio*) adj (disorder* or problem* or stress* or state* or trauma or consequence* or change* or disturbance* or outcome* or response*)) or ((community or individual* or people* or person*) adj3 (recovery or strength* or fortitude or resourceful* or adapt* or capacit* or vulnerabilit* or hardiness))).mp.
8	((capacit* adj2 adapt*) or (optimism* or pessimism* or optimistic* or pessimistic* or ((negative or positive or maladapt* or adaptive or poor*) adj (attitude* or behavio?r* or outlook or emotion* or reaction* or adjutment)))).mp.
9	(Sleep disturbance* or sleep disorder* or sleep problem* or nightmares or Insomnia* or phobias or (substance abuse or drug abuse or alcohol abuse or alcoholism)).mp.
10	5 or 6 or 7 or 8 or 9
11	homeless persons/ or homeless youth/ or vulnerable populations/ or working poor/ or social problems/ or poverty/ or minority groups/ or social isolation/ or social marginalization/ or poverty areas/ or unemployment/ or Cultural Deprivation/ or Medically Underserved Area/ or medical indigency/
12	american native continental ancestry group/ or indians, central american/ or indians, north american/ or alaskan natives/ or indigenous canadians/ or inuits/ or american natives/ or indians, south american/ or oceanic ancestry group/ or indigenous peoples/
13	(aborigin* or torres strait* or maori* or american indian* or inuit* or aleutian* or metis or ((Australian or New Zealand* or Canadian or American or Alaskan or Latin America* or South America) adj1 (native? or indigenous)) or (first people* or first nation*)).mp.
14	((Indigen* adj6 (communit* or resident* or perspective* or person* or people* or population or settlement* or village? or reservation? or reserve? or clan? or band? or confederac* or m#n or wom#n or boy? or girl? or male? or female? or adolescent? or youth? or adult? or child* or nation? or group? or tribe? or tribal)) or (native adj (communit* or settlement* or village? or

	reservation? or reserve? or clan? or band? or confederac* or nation? or people? or population? or m#n or wom#n or boy? or girl? or male? or female? or adolescent? or youth? or person? or adult? or child* or people* or tribe? or tribal or group?)) or ((tribe* or tribal) adj (communit* or settlement* or village? or reservation? or reserve? or clan? or band? or confederac* or people? or population? or m#n or wom#n or boy? or girl? or male? or female? or adolescent? or youth? or person? or adult? or child* or people* or nation? or group? or setting?))).mp.
15	(Disadvantaged or minorities or poverty or destitution or destitute or homeless* or marginali#ed or marginali#ation* or inequalities or inequities or Impoverish* or extremely poor or underpriv?leg* or unemployment or Latchkey Child or Runaways or Illiteracy or Underserved or Indigency or Indigent or ((Social* or community or inividual*) adj (vulnerab* or inequalit*))).mp.
16	(((minority or poor or poorer or racial or ethnic or vulnerable or linguistically diverse or culturally diverse or precarious) adj (communit* or population* or people or group* or setting* or background* or status* or settlement* or resident* or household* or societ* or demographic* or circumstance* or attribute* or condition*)) or ((socioeconomic* or socio-economic* or economic* or financial* or social* or cultural* or education* or housing) adj1 (disadvantage or deprivation or insecur* or precarity or precarious* or vulnerab* or hardship* or inequit* or disparit* or isolat* or adversity or instabilit* or unstable or deprivation* or discrimination*))).mp.
17	(((poor* or Informal* or unauthori#ed or vulnerab* or insecure* or precarious* or overcrowd* or over-crowd* or unstabl*) adj (home? or housing or residence* or settlement or housed)) or (slums or shanty towns or squats or pavement dweller* or ghetto* or "living on the streets" or living rough or street youth* or housing stress* or rental stress* or public housing)).mp.
18	(((ow* or lack* or "lack of" or limited or adverse or unstable or "having less" or diminished) adj (socioeconomic or socio-economic* or economic* or income or literacy or education* or median household or household or "level of education" or education level* or social capital or social support* or social connect* or money or finances)) or (working class* or blue collar worker* or migrant farm worker* or migrant worker* or low skill* or unskilled worker* or newly arrived migrant* or new migrant* or working poor or digital divide)).mp.
19	(((socioeconomic* or economic* or financial* or money or monetary) adj (challenge* or pressure* or strain* or stress* or cris#s)) or (socio* adj4 (gender or old age or ethnic* or racial* or older adult* or elderly or female* or women))).mp.
20	(disability status or "with disabilit*" or structural discrimination or ((single parent? or one parent or lone mother* or lone father* or young child*) and (family or families)) or ((lone or single or isolated) adj (person* or people))).mp.
21	or/11-20
22	cohort studies/ or follow-up studies/ or longitudinal studies/ or prospective studies/ or retrospective studies/ or cross-sectional studies/
23	(cohort stud* or cohort analys* or cross-sectional or (follow-up stud* or longitudinal stud* or prospective stud* or retrospective stud*)).mp.
24	grounded theory/ or qualitative research/ or focus groups/ or interviews as topic/ or "surveys and questionnaires"/ or self report/
25	(qualitative* or focus group* or case stud* or case research* or phenomenolog* or grounded theory or action research* or ethnograph* or thematic analys* or discourse analys* or narrative inquiry or narrative analys* or narrative research* or narrative stud* or conversation* analys* or biographical research* or biographical stud* or descriptive research or descriptive stud* or field research or survey* or questionnaire* or observation* research or autoethnograph* or participant observation* or structured observation* or participatory research or case series or interview*).mp.
26	(((personal or participant* or survivor* or respondent*) adj3 (story or stories or narrative* or journal* or diary or diaries or verbal response* or written response* or oral response*)) or (oral histor* or vignettes or solicited diaries or video diar* or audio diar* or oral testimon*)).mp.
27	22 or 23 or 24 or 25 or 26

28	4 and 10 and 21 and 27
29	limit 28 to english language

Embase Classic+Embase Search Strategy:

#	Searches
1	natural disaster/ or disaster/ or wildfire/ or earthquake/ or drought/ or tsunami/ or flooding/ or hurricane/ or forest fire/ or wildland fire/ or disaster victim/
2	(Bushfire* or bush fire* or wildfire* or wild fire* or Forest fire* or wildland fire* or wild land fire* or woodland fire* or wood land fire* or brushfire* or brush fire? or rural fire? or grassfire* or grass fire or vegetation fire? or disaster* or flooding or floods or earthquake* or earth quake* or hurricane* or tsunami* or cyclone* or typhoon* or drought? or Tornado* or avalanche* or landslides or volcanic eruption* or natural hazards or natural catastrophe* or ((disast* or catastroph*) adj (fire? or flood or tidal wave? or mudslide* or rockslide* or peatland fire* or peat fire* or storm* or blizzard*))).mp.
3	(postearthquake or postdisaster* or postflood* or post-flood or (Flood adj (related or experience* or exposure or trauma* or impact* or survivor*))).mp.
4	1 or 2 or 3
5	psychotrauma/ or mental disease/ or mental health/ or mental health care/ or mental health service/ or mental stress/ or depression/ or major depression/ or anxiety/ or anxiety disorder/ or psychological aspect/ or psychological resilience/ or psychological well-being/ or suicide/ or suicidal behavior/ or suicidal ideation/ or traumatic shock/ or posttraumatic stress disorder/ or psychology/ or coping behavior/ or wellbeing/ or distress syndrome/ or psychosocial care/ or "posttraumatic growth (psychology)"/ or survivorship/ or emotion/ or emotionality/ or adaptation/
6	(depressive* or mental* or post-traumatic stress* or Posttraumatic stress* or PTSD or PTSS or depression or anxiety or resilien* or coping or psych* or wellbeing or well-being or distress* or suicid* or self-harm or schizophreni* or mood disorder* or despair* or counsel?ing or (Posttraumatic growth or Post-traumatic growth)).mp.
7	(((emotional or behavio*) adj (disorder* or problem* or stress* or state* or trauma or consequence* or change* or disturbance* or outcome* or response*)) or ((community or individual* or people* or person*) adj3 (recovery or strength* or fortitude or resourceful* or adapt* or capacit* or vulnerabilit* or hardiness))).mp.
8	((capacit* adj2 adapt*) or (optimism* or pessimism* or optimistic* or pessimistic* or ((negative or positive or maladapt* or adaptive or poor*) adj (attitude* or behavio?r* or outlook or emotion* or reaction* or adjutment)))).mp.
9	(Sleep disturbance* or sleep disorder* or sleep problem* or nightmares or Insomnia* or phobias or (substance abuse or drug abuse or alcohol abuse or alcoholism)).mp.
10	5 or 6 or 7 or 8 or 9
11	homelessness/ or homeless person/ or homeless youth/ or vulnerable population/ or unemployment/ or lowest income group/ or poverty/ or medically underserved/ or minority group/ or working poor/ or social isolation/ or cultural deprivation/
12	indigenous people/ or alaska native/ or american indian/ or canadian aboriginal/ or first nation/ or indigenous australian/ or taiwanese aborigine/ or oceanic ancestry group/ or torres strait islander/ or "Maori (people)"/
13	(aborigin* or torres strait* or maori* or american indian* or inuit* or aleutian* or metis or ((Australian or New Zealand* or Canadian or American or Alaskan or Latin America* or South America) adj1 (native? or indigenous)) or (first people* or first nation*)).mp.
14	((Indigen* adj6 (communit* or resident* or perspective* or person* or people* or population or settlement* or village? or reservation? or reserve? or clan? or band? or confederac* or m#n or wom#n or boy? or girl? or male? or female? or adolescent?

	or youth? or adult? or child* or nation? or group? or tribe? or tribal)) or (native adj (communit* or settlement* or village? or reservation? or reserve? or clan? or band? or confederac* or nation? or people? or population? or m#n or wom#n or boy? or girl? or male? or female? or adolescent? or youth? or person? or adult? or child* or people* or tribe? or tribal or group?)) or ((tribe* or tribal) adj (communit* or settlement* or village? or reservation? or reserve? or clan? or band? or confederac* or people? or population? or m#n or wom#n or boy? or girl? or male? or female? or adolescent? or youth? or person? or adult? or child* or people* or nation? or group? or setting?))).mp.
15	(Disadvantaged or minorities or poverty or destitution or destitute or homeless* or marginali#ed or marginali#ation* or inequalities or inequities or Impoverish* or extremely poor or underpriv?leg* or unemployment or Latchkey Child or Runaways or Illiteracy or Underserved or Indigency or Indigent or ((Social* or community or inividual*) adj (vulnerab* or inequalit*))).mp.
16	(((minority or poor or poorer or racial or ethnic or vulnerable or linguistically diverse or culturally diverse or precarious) adj (communit* or population* or people or group* or setting* or background* or status* or settlement* or resident* or household* or societ* or demographic* or circumstance* or attribute* or condition*)) or ((socioeconomic* or socio-economic* or economic* or financial* or social* or cultural* or education* or housing) adj1 (disadvantage or deprivation or insecur* or precarity or precarious* or vulnerab* or hardship* or inequit* or disparit* or isolat* or adversity or instabilit* or unstable or deprivation* or discrimination*))).mp.
17	(((poor* or Informal* or unauthori#ed or vulnerab* or insecure* or precarious* or overcrowd* or over-crowd* or unstabl*) adj (home? or housing or residence* or settlement or housed)) or (slums or shanty towns or squats or pavement dweller* or ghetto* or "living on the streets" or living rough or street youth* or housing stress* or rental stress* or public housing)).mp.
18	(((ow* or lack* or "lack of" or limited or adverse or unstable or "having less" or diminished) adj (socioeconomic or socio-economic* or economic* or income or literacy or education* or median household or household or "level of education" or education level* or social capital or social support* or social connect* or money or finances)) or (working class* or blue collar worker* or migrant farm worker* or migrant worker* or low skill* or unskilled worker* or newly arrived migrant* or new migrant* or working poor or digital divide)).mp.
19	(((socioeconomic* or economic* or financial* or money or monetary) adj (challenge* or pressure* or strain* or stress* or cris#s)) or (socio* adj4 (gender or old age or ethnic* or racial* or older adult* or elderly or female* or women))).mp.
20	(disability status or "with disabilit*" or structural discrimination or ((single parent? or one parent or lone mother* or lone father* or young child*) and (family or families)) or ((lone or single or isolated) adj (person* or people))).mp.
21	or/11-20
22	cohort analysis/ or cross-sectional study/
23	(cohort stud* or cohort analys* or cross-sectional or (follow-up stud* or longitudinal stud* or prospective stud* or retrospective stud*)).mp.
24	ethnographic research/ or participatory research/ or qualitative research/ or phenomenology/ or case study/ or questionnaire/ or open ended questionnaire/ or structured questionnaire/ or interview/ or semi structured interview/ or structured interview/ or telephone interview/ or unstructured interview/ or self report/ or grounded theory/
25	(qualitative* or focus group* or case stud* or case research* or phenomenolog* or grounded theory or action research* or ethnograph* or thematic analys* or discourse analys* or narrative inquiry or narrative analys* or narrative research* or narrative stud* or conversation* analys* or biographical research* or biographical stud* or descriptive research or descriptive stud* or field research or survey* or questionnaire* or observation* research or autoethnograph* or participant observation* or structured observation* or participatory research or case series or interview*).mp.

26	(((personal or participant* or survivor* or respondent*) adj3 (story or stories or narrative* or journal* or diary or diaries or verbal response* or written response* or oral response*)) or (oral histor* or vignettes or solicited diaries or video diar* or audio diar* or oral testimon*)).mp.
27	22 or 23 or 24 or 25 or 26
28	4 and 10 and 21 and 27
29	limit 28 to english language
30	limit 29 to conference abstract
31	29 not 30

APA PsycInfo Search Strategy:

#	Searches
1	disasters/ or natural disasters/
2	(Bushfire* or bush fire* or wildfire* or wild fire* or Forest fire* or wildland fire* or wild land fire* or woodland fire* or wood land fire* or brushfire* or brush fire? or rural fire? or grassfire* or grass fire or vegetation fire? or disaster* or flooding or floods or earthquake* or earth quake* or hurricane* or tsunami* or cyclone* or typhoon* or drought? or Tornado* or avalanche* or landslides or volcanic eruption* or natural hazards or natural catastrophe* or ((disast* or catastroph*) adj (fire? or flood or tidal wave? or mudslide* or rockslide* or peatland fire* or peat fire* or storm* or blizzard*))).mp.
3	(postearthquake or postdisaster* or postflood* or post-flood or (Flood adj (related or experience* or exposure or trauma* or impact* or survivor*))).mp.
4	1 or 2 or 3
5	Anxiety/ or Anxiety Disorders/ or Major Depression/ or Mental Disorders/ or Mental Health/ or Mental Health Services/ or Posttraumatic Stress Disorder/ or Coping Behavior/ or Distress/ or Psychological Stress/ or Psychology/ or Well Being/ or Emotions/ or Counseling/ or Psychotherapy/ or Psychosocial Factors/ or Stress Reactions/ or Adaptation/ or Adjustment/ or "Depression (Emotion)"/ or "Resilience (Psychological)"/
6	(depressive* or mental* or post-traumatic stress* or Posttraumatic stress* or PTSD or PTSS or depression or anxiety or resilien* or coping or psych* or wellbeing or well-being or distress* or suicid* or self-harm or schizophreni* or mood disorder* or despair* or counsel?ing or (Posttraumatic growth or Post-traumatic growth)).mp.
7	(((emotional or behavio*) adj (disorder* or problem* or stress* or state* or trauma or consequence* or change* or disturbance* or outcome* or response*)) or ((community or individual* or people* or person*) adj3 (recovery or strength* or fortitude or resourceful* or adapt* or capacit* or vulnerabilit* or hardiness))).mp.
8	((capacit* adj2 adapt*) or (optimism* or pessimism* or optimistic* or pessimistic* or ((negative or positive or maladapt* or adaptive or poor*) adj (attitude* or behavio?r* or outlook or emotion* or reaction* or adjutment)))).mp.
9	(Sleep disturbance* or sleep disorder* or sleep problem* or nightmares or Insomnia* or phobias or (substance abuse or drug abuse or alcohol abuse or alcoholism)).mp.
10	5 or 6 or 7 or 8 or 9
11	disadvantaged/ or cultural deprivation/ or digital divide/ or economic disadvantage/ or social deprivation/ or homeless/ or social disadvantage/ or economic inequality/ or poverty/ or social isolation/ or lower income level/
12	minority groups/ or indigenous populations/ or alaska natives/ or american indians/ or inuit/ or hawaii natives/ or tribes/

13	(aborigin* or torres strait* or maori* or american indian* or inuit* or aleutian* or metis or ((Australian or New Zealand* or Canadian or American or Alaskan or Latin America* or South America) adj1 (native? or indigenous)) or (first people* or first nation*)).mp.
14	((Indigen* adj6 (communit* or resident* or perspective* or person* or people* or population or settlement* or village? or reservation? or reserve? or clan? or band? or confederac* or m#n or wom#n or boy? or girl? or male? or female? or adolescent? or youth? or adult? or child* or nation? or group? or tribe? or tribal)) or (native adj (communit* or settlement* or village? or reservation? or reserve? or clan? or band? or confederac* or nation? or people? or population? or m#n or wom#n or boy? or girl? or male? or female? or adolescent? or youth? or person? or adult? or child* or people* or tribe? or tribal or group?)) or ((tribe* or tribal) adj (communit* or settlement* or village? or reservation? or reserve? or clan? or band? or confederac* or people? or population? or m#n or wom#n or boy? or girl? or male? or female? or adolescent? or youth? or person? or adult? or child* or people* or nation? or group? or setting?))).mp.
15	(Disadvantaged or minorities or poverty or destitution or destitute or homeless* or marginali#ed or marginali#ation* or inequalities or inequities or Impoverish* or extremely poor or underpriv?leg* or unemployment or Latchkey Child or Runaways or Illiteracy or Underserved or Indigency or Indigent or ((Social* or community or inividual*) adj (vulnerab* or inequalit*))).mp.
16	(((minority or poor or poorer or racial or ethnic or vulnerable or linguistically diverse or culturally diverse or precarious) adj (communit* or population* or people or group* or setting* or background* or status* or settlement* or resident* or household* or societ* or demographic* or circumstance* or attribute* or condition*)) or ((socioeconomic* or socio-economic* or economic* or financial* or social* or cultural* or education* or housing) adj1 (disadvantage or deprivation or insecur* or precarity or precarious* or vulnerab* or hardship* or inequit* or disparit* or isolat* or adversity or instabilit* or unstable or deprivation* or discrimination*))).mp.
17	(((poor* or Informal* or unauthori#ed or vulnerab* or insecure* or precarious* or overcrowd* or over-crowd* or unstabl*) adj (home? or housing or residence* or settlement or housed)) or (slums or shanty towns or squats or pavement dweller* or ghetto* or "living on the streets" or living rough or street youth* or housing stress* or rental stress* or public housing)).mp.
18	(((ow* or lack* or "lack of" or limited or adverse or unstable or "having less" or diminished) adj (socioeconomic or socio-economic* or economic* or income or literacy or education* or median household or household or "level of education" or education level* or social capital or social support* or social connect* or money or finances)) or (working class* or blue collar worker* or migrant farm worker* or migrant worker* or low skill* or unskilled worker* or newly arrived migrant* or new migrant* or working poor or digital divide)).mp.
19	(((socioeconomic* or economic* or financial* or money or monetary) adj (challenge* or pressure* or strain* or stress* or cris#s)) or (socio* adj4 (gender or old age or ethnic* or racial* or older adult* or elderly or female* or women))).mp.
20	(disability status or "with disabilit*" or structural discrimination or ((single parent? or one parent or lone mother* or lone father* or young child*) and (family or families)) or ((lone or single or isolated) adj (person* or people))).mp.
21	or/11-20
22	Cohort Analysis/ or longitudinal studies/ or prospective studies/ or followup studies/ or retrospective studies/
23	(cohort stud* or cohort analys* or cross-sectional or (follow-up stud* or longitudinal stud* or prospective stud* or retrospective stud*)).mp.
24	qualitative methods/ or focus group/ or grounded theory/ or interpretative phenomenological analysis/ or narrative analysis/ or semi-structured interview/ or thematic analysis/ or phenomenology/ or qualitative measures/ or focus group interview/ or group discussion/ or participant observation/ or direct observation/
25	(qualitative* or focus group* or case stud* or case research* or phenomenolog* or grounded theory or action research* or ethnograph* or thematic analys* or discourse analys* or narrative inquiry or narrative analys* or narrative research* or narrative

	stud* or conversation* analys* or biographical research* or biographical stud* or descriptive research or descriptive stud* or field research or survey* or questionnaire* or observation* research or autoethnograph* or participant observation* or structured observation* or participatory research or case series or interview*).mp.
26	(((personal or participant* or survivor* or respondent*) adj3 (story or stories or narrative* or journal* or diary or diaries or verbal response* or written response* or oral response*)) or (oral histor* or vignettes or solicited diaries or video diar* or audio diar* or oral testimon*)).mp.
27	22 or 23 or 24 or 25 or 26
28	4 and 10 and 21 and 27
29	limit 28 to english language

CINAHL Search Strategy

#	Query
S29	S23 OR S27<br>Limiters - English Language
S28	S23 OR S27
S27	S4 AND S10 AND S19 AND S26
S26	S24 OR S25
S25	((qualitative* or "focus group*" or "case stud*" or "case research*" or phenomenolog* or "grounded theory" or "action research*" or ethnograph* or "thematic analys*" or "discourse analys*" or "narrative inquiry" or "narrative analys*" or "narrative research*" or "narrative stud*" or "conversation* analys*" or "biographical research*" or "biographical stud*" or "descriptive research" or "descriptive stud*" or "field research" or survey* or questionnaire* or "observation* research" or autoethnograph* or "participant observation*" or "structured observation*" or "participatory research" or "case series" or interview*)) OR (((personal or participant* or survivor* or respondent*) N3 (story or stories or narrative* or journal* or diary or diaries or narrative* or "verbal response*" or "written response*" or "oral response*"))) OR (("oral histor*" or vignettes or "solicited diaries" or "video diar*" or "audio diar*" or "oral testimon*"))
S24	(MH "Qualitative Studies") OR (MH "Action Research") OR (MH "Ethnographic Research") OR (MH "Ethnological Research") OR (MH "Grounded Theory") OR (MH "Naturalistic Inquiry") OR (MH "Phenomenological Research") OR (MH "Phenomenology")
S23	S4 AND S10 AND S19 AND S22
S22	S20 OR S21
S21	(("cohort stud*" or "cohort analys*" or "cross-sectional")) OR (("follow-up stud*" or "longitudinal stud*" or "prospective stud*" or "retrospective stud*"))
S20	(MH "Prospective Studies") OR (MH "Pseudolongitudinal Studies") OR (MH "Cross Sectional Studies") OR (MH "Retrospective Design")
S19	S11 OR S12 OR S13 OR S14 OR S15 OR S16 OR S17 OR S18
S18	((socio* N4 (gender or "old age" or ethnic* or racial* or "older adult*" or elderly or female* or women))) OR (("disability status" or “with disabilit*” or "structural discrimination")) OR ((("single parent?" or "one parent" or "lone mother*" or "lone father*" or "young child*") and (family or families))) OR (((lone or single or isolated) N0 (person* or people)))
S17	(((ow* or lack* or "lack of" or limited or adverse or unstable or "having less" or diminished) N0 (socioeconomic or "socio-economic*" or economic* or income or literacy or education* or "median household" or household or "level of education" or "education level*" or "social capital" or "social support*" or "social connect*" or money or finances))) OR (("working class*" or "blue collar worker*" or "migrant farm worker*" or "migrant worker*" or "low skill*" or "unskilled worker*" or "newly arrived migrant*" or "new migrant*" or "working poor" or "digital divide")) OR (((socioeconomic* or economic* or financial* or money or monetary) N0 (challenge* or pressure* or strain* or stress* or cris#s)))
S16	(((socioeconomic* or "socio-economic*" or economic* or financial* or social* or cultural* or education* or housing) N1 (disadvantage or deprivation or insecur* or precarity or precarious* or vulnerab* or hardship* or inequit* or disparit* or isolat* or adversity or instabilit* or unstable or deprivation* or discrimination* or problems))) OR (((poor* or Informal* or unauthori#ed or vulnerab* or insecure* or precarious* or overcrowd* or "over-

	crowd*" or unstabl*) N0 (home? or housing or residence* or settlement or housed))) OR ((slums or "shanty towns" or squats or "pavement dweller*" or ghetto* or "living on the streets" or "living rough" or "street youth*" or "housing stress*" or "rental stress*" or "Public Housing"))
S15	((Disadvantaged or minorities or poverty or destitution or destitute or homeless* or marginali#ed or marginali#ation* or inequalities or inequities or Impoverish* or "extremely poor" or underpriv?leg* or unemployment or "Latchkey Child*" or Runaways or Illiteracy or Underserved or Indigency or Indigent)) OR (((Social* or community or inividual*) N0 (vulnerab* or inequalit*))) OR (((minority or poor or poorer or racial or ethnic or vulnerable or "linguistically diverse" or "culturally diverse" or precarious) N0 (communit* or population* or people or group* or setting* or background* or status* or settlement* or resident* or household* or societ* or demographic* or circumstance* or attribute* or condition*)))
S14	((Indigen* N6 (communit* or resident* or perspective* or person* or people* or population or settlement* or village? or reservation? or reserve? or clan? or band? or confederac* or m#n or wom#n or boy? or girl? or male? or female? or adolescent? or youth? or adult? or child* or nation? or group? or tribe? or tribal))) OR ((native N0 (communit* or settlement* or village? or reservation? or reserve? or clan? or band? or confederac* or nation? or people? or population? or m#n or wom#n or boy? or girl? or male? or female? or adolescent? or youth? or person? or adult? or child* or people* or tribe? or tribal or group?))) OR (((tribe* or tribal) N0 (communit* or settlement* or village? or reservation? or reserve? or clan? or band? or confederac* or people? or population? or m#n or wom#n or boy? or girl? or male? or female? or adolescent? or youth? or person? or adult? or child* or people* or nation? or group? or setting?)))
S13	((aborigin* or "torres strait*" or maori* or "american indian*" or inuit* or aleutian* or metis)) OR (((Australian or "New Zealand*" or Canadian or American or Alaskan or "Latin America*" or "South America*") N1 (native? or indigenous))) OR (("first people*" or "first nation*"))
S12	(MH "Indigenous Peoples") OR (MH "Aboriginal Canadians") OR (MH "First Nations of Canada") OR (MH "Arctic Peoples") OR (MH "Inuit") OR (MH "First Nations of Australia") OR (MH "Aboriginal Australians") OR (MH "Torres Strait Islanders") OR (MH "Maori") OR (MH "Native Americans") OR (MH "Alaska Natives") OR (MH "Indigenous Health")
S11	(MH "Homeless Persons") OR (MH "Homelessness") OR (MH "Latchkey Children") OR (MH "Social Problems") OR (MH "Poverty") OR (MH "Runaways") OR (MH "Public Housing") OR (MH "Medically Underserved Area") OR (MH "Vulnerability") OR (MH "Social Isolation") OR (MH "Indigent Persons") OR (MH "Minority Groups") OR (MH "Illiteracy") OR (MH "Poverty Areas") OR (MH "Medically Underserved") OR (MH "Medically Uninsured")
S10	S5 OR S6 OR S7 OR S8 OR S9
S9	(("Sleep disturbance*" or "sleep disorder*" or "sleep problem*" or nightmares or Insomnia* or phobias)) OR (("substance abuse" or "drug abuse" or "alcohol abuse" or alcoholism))
S8	(((community or individual* or people* or person*) N3 (recovery or strength* or fortitude or resourceful* or adapt* or capacit* or vulnerabilit* or hardiness))) OR (capacit* N2 adapt*) OR ((optimism* or pessimism* or optimistic* or pessimistic*)) OR (((negative or positive or maladapt* or adaptive or poor*) N0 (attitude* or behavio?r* or outlook or emotion* or reaction* or adjutment)))
S7	((depressive* or mental* or "post-traumatic" or Posttraumatic* or PTSD or PTSS or depression or anxiety or resilien* or coping or psych* or wellbeing or "well-being" or distress* or suicid* or "self-harm" or schizophreni* or "mood disorder*" or despair* or counsel?ing)) OR (("Posttraumatic growth" or "Post-traumatic growth")) OR (((emotional or behavio*) N0 (disorder* or problem* or stress* or state* or trauma or consequence* or change* or disturbance* or outcome* or response*)))
S6	(MH "Counseling") OR (MH "Psychology, Applied") OR (MH "Mental Health Services+") OR (MH "Coping") OR (MH "Symptom Distress") OR (MH "Adaptation, Psychological") OR (MH "Posttraumatic Growth, Psychological") OR (MH "Psychosocial Functioning") OR (MH "Support, Psychosocial") OR (MH "Hardiness") OR (MH "Stress") OR (MH "Stress, Psychological") OR (MH "Anxiety+") OR (MH "Depression") OR (MH "Emotional Lability") OR (MH "Affective Symptoms") OR (MH "Mental Status") OR (MH "Survivorship") OR (MH "Mental Health") OR (MH "Suicide") OR (MH "Suicidal Ideation") OR (MH "Suicide, Attempted")
S5	(MH "Mental Disorders") OR (MH "Adjustment Disorders+") OR (MH "Mental Disorders, Chronic") OR (MH "Neurotic Disorders") OR (MH "Affective Disorders+") OR (MH "Anxiety Disorders+") OR (MH "Dissociative Disorders+") OR (MH "Organic Mental Disorders") OR (MH "Personality Disorders+") OR (MH "Psychophysiologic Disorders") OR (MH "Psychotic

	Disorders") OR (MH "Affective Disorders, Psychotic+") OR (MH "Paranoid Disorders") OR (MH "Schizoaffective Disorder") OR (MH "Schizophrenia+") OR (MH "Psychological Trauma")
S4	S1 OR S2 OR S3
S3	(((disast* or catastroph*) N0 (fire* or flood or "tidal wave?" or mudslide* or rockslide* or "peatland fire*" or "peat fire*" or storm* or blizzard*))) OR ((postearthquake or postdisaster* or postflood* or "post-flood")) OR ((Flood adj (related or experience* or exposure or trauma* or impact* or survivor*)))
S2	(Bushfire* or "bush fire*" or wildfire* or "wild* fire*" or "Forest fire*" or "wildland fire*" or "wild* land fire*" or "woodland fire*" or "wood land fire*" or brushfire* or "brush fire?" or "rural fire?" or grassfire* or "grass fire" or "vegetation fire?" or disaster* or flooding or floods or earthquake* or "earth quake*" or hurricane* or tsunami* or cyclone* or typhoon* or drought? or Tornado* or avalanche* or landslides or "volcanic eruption*" or "natural hazards" or "natural catastrophe*")
S1	(MH "Disasters") OR (MH "Fires") OR (MH "Wildfires") OR (MH "Natural Disasters")
